# Mitochondrial Control for Healthy and Autoimmune T Cells

**DOI:** 10.3390/cells12131800

**Published:** 2023-07-07

**Authors:** Li Jia, Lei Zhang, Mengdi Liu, Huiyan Ji, Zhenke Wen, Chunhong Wang

**Affiliations:** 1 Jiangsu Key Laboratory of Infection and Immunity, Institutes of Biology and Medical Sciences, Soochow University, Suzhou 215123, China; 2 Cyrus Tang Hematology Center, State Key Laboratory of Radiation Medicine and Protection, Soochow University, Suzhou 215123, China

**Keywords:** T cells, autoimmunity, mitochondria, metabolic adaptation

## Abstract

T cells are critical players in adaptive immunity, driving the tissue injury and organ damage of patients with autoimmune diseases. Consequently, investigations on T cell activation, differentiation, and function are valuable in uncovering the disease pathogenesis, thus exploring promising therapeutics for autoimmune diseases. In recent decades, accumulating studies have pinpointed immunometabolism as the fundamental determinant in controlling T cell fate. Specifically, mitochondria, as a hub of intracellular metabolism, connect glucose, lipid, and amino acid metabolic pathways. Herein, we summarize metabolic adaptations of mitochondrial oxidative phosphorylation and the relevant glucose, lipid, and amino acid metabolism during T cell activation, differentiation, and function. Further, we focused on current updates of the molecular bases for metabolic reprogramming in autoimmune T cells and advances in exploring metabolic-targeted therapeutics against autoimmune diseases. This might facilitate the in-depth understanding of autoimmune pathogeneses and the clinical management of autoimmune diseases.

## 1. Mitochondrial Dynamics Determine T Cell Fate

The immune system can distinguish between “self” and “non-self,” producing an effective immune response to non-self-antigens and a no/weak response to self-antigens, called immune tolerance. However, when the immune tolerance is broken, or the autoimmune cell regulation is abnormal under some internal and external causes, such as genetic factors and environmental factors, the immune system will produce a continuous immune response to self-antigens [1,2], resulting in tissue injury and autoimmune diseases.

CD4 T cells are helper T cells that connect innate and adaptive immunity [3], licensing subsequent cellular and humoral immunity and thus playing a central role in the immunopathology of autoimmune diseases [3,4,5]. They differentiate into T-helper 1 (Th1), T-helper 2 (Th2), T-helper 17 (Th17), follicular helper T (Tfh), and regulatory T (Treg) cells [6], exerting non-redundant functions in autoimmunity [6,7,8]. Thus, mechanistic investigations underpinning the differentiation and function of T cells in autoimmune diseases are crucial for elucidating autoimmune immunopathology and exploring therapeutic strategies in clinical practice.

Metabolic reprogramming affects the development of autoimmune diseases by regulating T cells’ differentiation and function [9,10]. Of note, immune metabolism shares a common metabolic pathway that produces adenosine triphosphate (ATP) through the tricarboxylic acid (TCA) cycle within mitochondria [11]. Specifically, glucose needs to be broken down to pyruvate before entering the mitochondria; at this point, anaerobic respiration can occur to produce ATP, but with a relatively low efficiency [12]. Pyruvate must first be converted to acetyl coenzyme A (acetyl-CoA) through an enzymatic reaction. After that, acetyl CoA enters the TCA cycle, is oxidized, loses electrons, and produces carbon dioxide—the loss of electrons from the acetyl CoA shuttle between many molecules within the mitochondria [11]. Moving electrons within the mitochondria causes electron fluctuations, creating a chemical gradient that drives the production of ATP, a process called oxidative phosphorylation. The ATP produced is transported outside the mitochondria so the cell can use it to carry out thousands of biochemical reactions.

**Figure 1:**
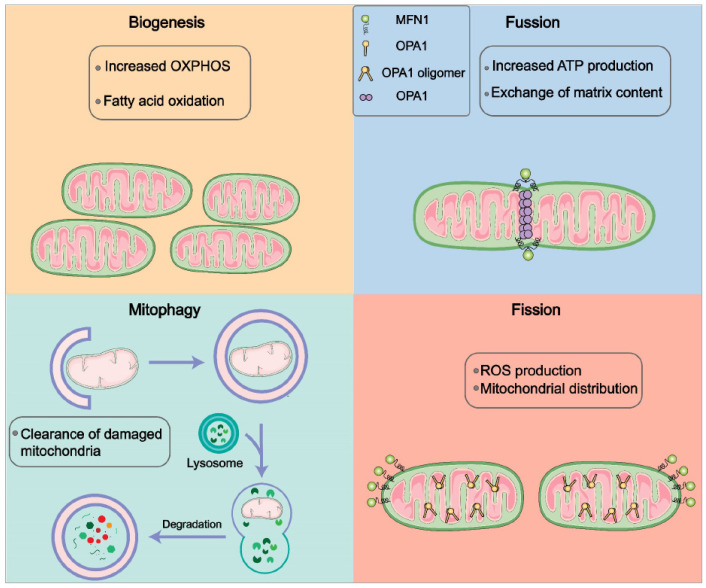
Mitochondrial dynamics. Mitochondria are highly dynamic organelles, constantly undergoing biogenesis, fusion, fission, and mitophagy. In response to developmental signals, cells initiate mitochondrial biogenesis via self-renewal. With the involvement of mitochondrial-shaping proteins, including OPA1, MFN1, and MFN2, mitochondria fusion by merging the outer and inner membranes shows elongated mitochondria with robust OXPHOS and ATP production. Meanwhile, cells maintain high-quality mitochondria by removing the dysfunctional or damaged ones via mitochondrial fission and subsequent mitophagy.

Most of the energy cells need is produced by mitochondria, essential organelles in cell energy metabolism. Mitochondrial membranes maintain mitochondrial function by altering the crystal structure [13], respiratory complex organization, mitochondrial DNA metabolism, and organelle distribution [13]. Thus, mitochondrial function is tightly related to its morphology, termed mitochondrial dynamics. Specifically, mitochondrial biogenesis, fusion, fission, and mitophagy are continuously occurring processes for maintaining homeostasis (Figure 1).

### 1.1. Mitochondrial Fusion in T Cells

Mitochondrial fusion has been increasingly implicated in T cell fate and memory development. Optic atrophy protein 1 (OPA1) [14], a mitochondrial shaping protein that controls crista biogenesis and respiration, is required for memory T cell function [14]. Specific small ubiquitin-like modifier (SUMO) protease 1 (SENP1) can activate Sirtuins 3 (Sirt3) deacetylase activity in T cells’ mitochondria, resulting in a decrease in the acetylation level of mitochondrial metalloprotease YME1 Like 1 ATPase (YME1L1) [14]. Deacetylation of YME1L1 inhibits its activity on OPA1 cleavage, thereby promoting mitochondrial fusion, leading to T cells’ survival and promoting T cell memory development [14]. Meanwhile, glycolytic intermediate fructose-1,6-bisphosphate (FBP) acts as a negative regulator, restricting the activation of SENP1-Sirt3 by AMP-activated protein kinase (AMPK) [14]. These findings suggest that glucose-limited activation of AMPK and subsequent SENP1-Sirt3 signaling promotes mitochondrial fusion and the development of T cell memory [14].

There is evidence that mitochondrial fusion plays a role in T cell differentiation. Specifically, primitive CD4 T (TN) cells expressing mitochondria-targeting fluorescent proteins can be polarized into Th1, Th2, Th17, and Treg subsets, in which Th17 cells contain elongated mitochondria and tight cristae, unlike other T cell subsets [15]. Among them, OPA1 regulates mitochondrial ridge morphology and fusion. When OPA1 is absent, it amplifies glutamine oxidation, resulting in an impaired NADH/NAD+ balance and accumulation of TCA cycle metabolites and 2-hydroxyglutarate, which negatively affects interleukin-17A (IL-17A) expression and Th17 cell differentiation [15].

In addition to playing an essential role in the memory formation and differentiation of T cells, mitochondrial fusion is also critical for the maturation of thymocytes in the double negative (DN) 3 stage. Analyzing mitochondrial function at different stages of thymocyte maturation when the T cell receptor β (TCR-β) site is rearranged, an oxidative phosphorylation-dependent process occurs at the DN3 stage of T cell development [16]. OPA1 depletion impairs respiration and reduces the number of DN3 cells during early T cell development. OPA1-deficient DN3 cells show more vital TCR signaling and are more prone to cell death [16]. Surviving OPA1^−/−^ thymocytes reach the periphery as mature T cells [16], displaying an effector memory phenotype even without antigenic stimulation, but cannot generate metabolically matched long-term memory T cells.

### 1.2. Mitochondrial Fission in T Cells

Mitochondrial fission is a mitochondrial fragmentation process regulated by associated proteins such as DNML (yeast) or dynamin-related protein 1 (DRP1) (mammalian cells) and is an essential feature of mitochondrial dynamics [17]. DRP1 is the main protein involved in mitochondrial fission, and its activity is tightly regulated so that the mitochondrial shape is strictly controlled following the needs of the cell [17,18]. DRP1 has been shown to generate the correct number of thymocytes by influencing thymocyte development, regulating effector T cell numbers in vivo, and maintaining T cell clonal expansion in vitro [19]. Disruption of mitochondrial fission during T cell development results in decreased thymocyte and mature T cell numbers and a reduced migration of mature circulating T cells to secondary lymphoid organs [20]. Consequently, altered mitochondrial fission is closely associated with disease development.

While effector memory T cells’ mitochondria maintain a fusion network, effector T cells have punctate mitochondria [21]. Mitochondrial fusion in effector memory T cells resets the electron transfer chain complex (ETC), favoring the oxidative phosphorylation system (OXPHOS) and fatty acid oxidation (FAO) by altering the cristae morphology of mitochondria. In contrast, mitochondrial fission in effector T cells causes cristae swelling, reducing ETC efficiency and promoting aerobic glycolysis [21]. Effector memory T cells have more mitochondrial mass than effector T cells, which exert lower levels of mitofusin 2 (MFN2) and OPA1 than effector memory T cells [21]. Of note, metformin can reduce mitochondrial fission by inhibiting S6 phosphorylation (p-S6) in the mammalian target of the rapamycin (mTOR) pathway and increase superoxide production in T cells, directly regulating T cell effector responses [22].

### 1.3. Mitophagy in T Cells

Mitophagy is a selective process for removing damaged or burnt-out mitochondria, which is fundamental in avoiding the overproduction of reactive oxygen species (ROS) [23]. Accumulating evidence has assigned an essential function of mitophagy in autoimmune diseases. As such, defects of lysosomal parts in Treg cells impair the completion of mitophagy, exacerbating mitochondrial oxidative stress and leading to Treg cell death in autoimmunity [24]. OXPHOS Th17 cells exhibit an increased mitochondrial fitness and an anti-apoptotic phenotype marked by high BCL-XL and low BIM [25]. In contrast, glycolytic Th17 cells show more mitophagy and an imbalance in BCL-XL to BIM [25]. USP30, a deubiquitinating enzyme on the outer membrane of mitochondria, can inhibit ubiquitination and prevent mitophagy [26]. Thus, the number of mitochondria is reduced, and the primary oxidative phosphorylation function is significantly impaired in most T cells from USP30-null mice. Meanwhile, the total ATP level and behaviors such as motility, polarization, signaling, and secretion of T cells were not disturbed in USP30-null mice [26]. However, the function of USP30-deficient T cells is affected by the impairment of the translation process of perforin, granzyme B, IFN-γ, TNF-α, and other cytokines [26]. In the collective, mitophagy is critically involved in T cell functions.

Mitochondrial dynamics are critical for T cell development in the thymus and for T cell differentiation after maturation. OPA1 is a mitochondrial shaping protein, and DRP1 is a protein involved in mitochondrial fission, which can affect the fusion and fission of mitochondria, leading to changes in the development and differentiation of T cells. In addition, mitochondrial mitophagy also plays a vital role in autoimmune diseases [27]. Both impaired and enhanced mitochondrial mitophagy can regulate the oxidative phosphorylation of T cells, thereby interfering with the differentiation and function of T cells.

## 2. Mitochondrial Control of Glucose Metabolism in T Cells

**Figure 2:**
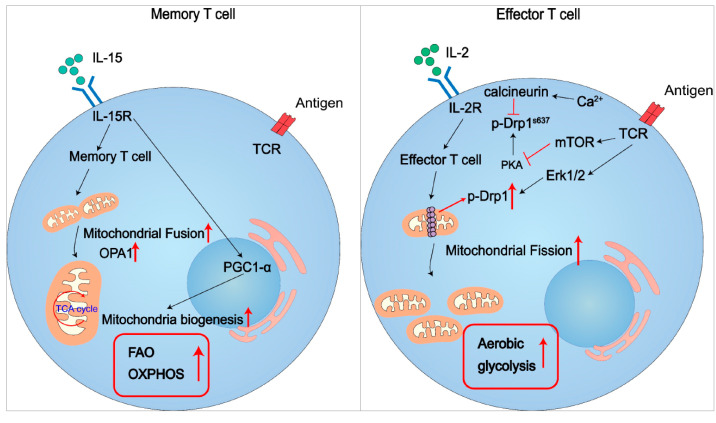
Metabolic adaptation determines the T cell fate. Mitochondrial fusion and fission control the memory and effector function of T cells. Memory T cells are characterized by an elevated expression of OPA1 and hyper-activity of mitochondrial fusion, relying on fatty acid oxidation(FAO) and mitochondrial OXPHOS. In contrast, effector T cells exert robust activity of DRP1, driving mitochondrial fission and thus depending on glycolysis to meet the energy needs. In conclusion, memory T cells have enhanced FAO and OXPHOS, and effector T cells exhibit a high rate of aerobic glycolysis.

In both in vivo and in vitro, activated T cells exhibit higher levels of intracellular acidification rates (ECARs) and oxygen consumption rates (OCRs) compared to naive T cells [28], suggesting that activated T cells simultaneously use aerobic glycolysis and OXPHOS. Meanwhile, different T cell subsets exert different mitochondrial metabolism pathways [29,30,31]. For example, Th1/Th2/Th17 cells maintain their functions mainly through aerobic glycolysis, while Treg development primarily depends on FAO within mitochondria [28]. CD4 effector memory T cells depend primarily on glycolysis, while CD8 effector memory T cells rely on FAO. Therefore, understanding the nature of mitochondrial metabolism and how it is regulated is vital to understand how immune cells are reprogrammed [29,30,31] (Figure 2).

### 2.1. Glycolysis in T Cells

Glucose catabolism can be divided into aerobic oxidation, anaerobic oxidation, and pentose phosphate pathways, depending on the metabolic characteristics and oxygen supply of different types of cells [32]. In aerobic oxidation, glucose is oxidized to pyruvate in sufficient oxygen. The pyruvate is oxidized to acetyl-CoA, which is further oxidized to water and carbon dioxide via the TCA cycle in mitochondria. Another condition is that, in the absence of oxygen, pyruvate is catalyzed by lactate dehydrogenase to produce lactate and 2 moles of ATP, which is known as anaerobic glycolysis. In addition, with insufficient oxygen, some cells still metabolize glucose to pyruvate to produce lactate, known as the Warburg effect [32]. The pentose phosphate pathway (PPP) is a metabolic pathway that branches from glucose-6-phosphate, creating ribose-5-phosphate for generating nucleic acids and nicotinamide adenine dinucleotide phosphate (NADPH) for the production of fatty acids, nucleotides, and nonessential amino acids.

At rest, T cells function primarily by oxidative phosphorylation, independent of glycolysis. However, effector T cells’ proliferation, differentiation, and survival require glycolysis [33]. Glucose transporter 1 (GLUT1) can increase glucose uptake and have a high expression in Th1, Th2, and Th17 cells. It supports the differentiation and proliferation of T cells by promoting glucose metabolism [34,35]. Pyruvate dehydrogenase (PDH) is located in the inner mitochondrial membrane. It enables the irreversible decarboxylation of pyruvate, producing acetyl-CoA. A metabolic analysis shows that PDH [36], whose function is inhibited by PDH kinases (PDHKs), is a critical checkpoint between glycolysis and oxidative metabolism in T cells. PDHK1 is expressed in Th17 but not in Th1 cells and is less defined in Treg cells [36]. Inhibited or knockout PDHK1 expression selectively inhibits Th17 cells and increases the proportion of Treg. Therefore, targeting specific T cell groups by regulating the balance of glycolysis and oxidative metabolism is a promising strategy for treating autoimmune diseases [36].

The glycolytic enzyme phosphoglycerate alterase (Pgam1) catalyzes the conversion of 3-phosphoglycerate (3-PG) to 2-phosphoglycerate and can regulate glycolysis and biosynthesis. Pgam1-deficient T cells have reduced glycolysis compared with normal cells. Th1, Th2, and Th17 differentiation is impaired in Pgam1-deficient T cells, whereas the Treg cell generation is only marginally affected. Not only CD4 T cells but also CD8 T cells are regulated by Pgam1. Pgam1-deficient CD8 T cells have a reduced capacity to produce IL-2, IFN-γ, and TNF-α, suggesting that glycolysis is required for effector differentiation of CD8 T cells. Augmenting glycolytic flux also drives CD8 T cells toward a terminally differentiated state, while its inhibition preserves the formation of long-lived memory CD8 T cells [33].

The role of glycolysis in Treg cells has yet to be unified and remains controversial. It has been found that the expression of GLUT1 on the surface of Treg cells is low, and the generation and expansion of Treg cells do not depend on glycolysis [37]. In GLUT1 transgenic mice, Treg cells have activated AMPK and are dependent on lipid oxidation [37,38]. The Treg transcription factor Foxp3 re-encodes T cell metabolism by inhibiting Myc and glycolysis, enhancing oxidative phosphorylation, and increasing nicotinamide adenine dinucleotide (NAD) oxidation, enabling Treg to function in a low-glucose, high-lactate environment [39]. As a result, glycolysis is less involved in Treg function, and oxidative metabolism is required [40]. However, studies have also shown that the inductive and inhibitory processes of regulatory T (iTreg) cells are closely dependent on glycolysis. Enolase-1, a glycolytic enzyme that binds to regulatory regions of Foxp3, like the promoter and conserved non-coding sequence 2 (CNS2), directly regulates the generation of iTreg cells [41]. In addition, in vitro experiments report that human Treg cells exhibit high glycolysis and require glycolysis to achieve their proliferation and inhibitory functions [41].

### 2.2. Mitochondrial Respiration in T Cells

When resting, T cells rely on mitochondria respiration and FAO to reduce the production of toxic metabolites that may damage cell function [42]. After T cells are activated, glycolysis increases but continues to produce the ATP required for T cell activity through aerobic respiration. At the same time, a carbon metabolic pathway is induced to meet the needs of rapid proliferation and synthetic metabolism. Acetyl-CoA is essential in promoting T cell activation, while alpha-ketoglutaric acid is an integral part of the demethylation reaction [43].

Effector memory T cells have minimal metabolic needs at this stage, mainly through FAO and mitochondrial respiration. Meanwhile, during the activation of T cells, mitochondrial respiration also leads to the production of ROS [35], which stimulates the production of crucial transcription factors NFAT for early activation of T cells [44]. In addition, ROS directly inhibits oxygen-dependent histone demethylase, which is essential for T cell metabolic reprogramming.

Differentiation of T cells from the naive state requires a shift from oxidative phosphorylation to aerobic glycolysis. Glycolysis is now known to be needed for effector differentiation of Th1, Th2, Th17, and CD8 T cells. However, the role of glycolysis in Tregs has yet to be unified. One theory holds that the generation and amplification of Treg cells do not depend on glycolysis, and oxidative phosphorylation is important. Another theory holds that glycolysis is necessary for the proliferation and function of Treg. Therefore, the metabolic recombination mechanism of Treg differentiation needs to be further studied.

## 3. Mitochondrial Control of Lipid Metabolism in T Cells

In addition to glucose, fatty acid metabolism is essential during T cell activation and differentiation [45,46]. Fatty acid metabolism includes anabolism that occurs in the cytoplasm and catabolism that mainly occurs in mitochondria [47]. Lipids and fatty acids are essential components of cell membranes and are necessary for a multitude of cellular functions, including membrane construction, signal transduction, and other biological activities [48,49,50].

### 3.1. Lipogenesis in T Cells

Lipogenesis includes the synthesis of both saturated and unsaturated fatty acids. The first step in synthesizing saturated fatty acids is the transport of acetyl-CoA from the mitochondria to the cytoplasm. Acetyl-CoA mainly comes from a product of glycolysis; when pyruvate enters the mitochondria, it will produce acetyl-CoA, which crosses the mitochondrial membrane and enters the cytosol. In addition, citric acid in the TCA cycle can transport to the cytosol and then release acetyl-CoA under the action of citrate synthetase. After that, under the catalysis of acetyl-CoA carboxylase, it will generate malonyl-CoA, and the carbon chain of fatty acid, which includes the cycle of condensation, reduction, dehydration, and reduction, is continuously extended.

Recent studies have shown that in addition to increased glucose metabolism, activated T cells also experience increased lipid synthesis [51]. Specifically, proliferating T cells must be fed various cell processes, including membrane synthesis, ATP production, and signal transduction. Th17 cells can efficiently synthesize lipids, fatty acid synthase (FASN), and a multi-enzyme complex from glucose to regulate the conversion of acetyl-CoA and malonyl-Coase into saturated long-chain fatty acids to control the differentiation and function of Th17 cells [52,53].

### 3.2. Lipid Oxidation in T Cells

Oxidation of fatty acids mainly occurs in mitochondria, providing energy to generate ATP, especially when circulating glucose concentrations are low. The fatty acids enter into mitochondria that mediate FAO and are situated in the outer membrane. Still, as the mitochondria cannot oxidize very long chain fatty acids [54], these are preferably metabolized via peroxisomal β-oxidation. The FAO begins by activating fatty acid, which is fatty acid uptake into the cell, esterification to CoA, and transport into mitochondria. One molecule of palmitic acid has seven carbon atoms, which undergo seven-times β-oxidation to produce seven molecules of flavin adenine dinucleotide 2 (FADH2) and eight molecules of acetyl-CoA; all of these molecules can generate 108 mol ATP through the ETC. Therefore, mitochondria are the primary energy production center and are crucial in fatty acid metabolism, affecting the differentiation and function of T cells [55].

Lipid oxidation provides energy requirements for cells and raw materials for cell growth, which is a fundamental element in maintaining life and homeostasis [56]. On the one hand, T cell expansion requires the synthesis of cell membranes and organelle membranes, which requires a large amount of ATP. At the same time, a fatty acid can supply energy through β-oxidation in mitochondria. On the other hand, lipid changes can regulate the differentiation and function of T cells, especially Treg cells [56]. Treg cells are shown to optimize lipids at a high rate and can expand and be functional in the absence of glucose. Regulation of the Raptor/mTORC1 signaling pathway in Treg cells can enhance cholesterol and lipid metabolism [57]. Of note, the mevalonate pathway is specifically crucial in orchestrating Treg cell proliferation and upregulating the suppressor molecules cytotoxic T-lymphocyte antigen-4 (CTLA4) and Inducible Co-Stimulator (ICOS) to establish Treg cell function, playing a central role in immune homeostasis [58]. In addition, fatty acid binding proteins (FABPs), which boost the uptake and transport of intracellular lipids, are a class of lipid chaperone proteins [59]. Inhibition of FABP5 in Treg triggers the release of mitochondrial DNA (mtDNA) and the subsequent transduction of cGAS-STING-dependent type I IFN signaling, inducing the regulatory cytokine IL-10 and promoting the inhibitory activity of Treg cells [59], which attenuates autoimmune diseases [60].

## 4. Mitochondrial Control of Amino Acid Metabolism in T Cells

Amino acid metabolism mainly occurs in mitochondria. Most amino acid deamination occurs via oxidative deamination of glutamic acid. Then, glutamic acid is transported into mitochondria for the ornithine cycle, which synthesizes ammonia and CO2 into urea and generates a molecule of fumaric acid. Amino acids are not only the cornerstone of building protein polypeptide chains but also important regulatory factors in the processes of cell metabolism and protein translation and proliferation. The catabolism pathway of amino acids is a crucial checkpoint for immunity [61]. In T cell activation, differentiation, and function, amino acids are energy sources and substrates for protein and nucleic acid biosynthesis [61]. Increasing the expression of glucose and amino acid transporters increases the uptake rate of these nutrients. Glutamine is important to T cell activation, and the activated T cells have a 5–10-fold higher rate of glutamine uptake. [62]. Glutamine deficiency impairs T cell activation events, including proliferation and cytokine secretion [63].

### 4.1. Amino Acid Transporters on T Cells

Amino acid (AA) transporters are crucial for T cell-mediated adaptive immunity. L-type amino acid transporters (LAT1), alanine serine cysteine preferring transporter 2 (ASCT2), γ-aminobutyric acid (GABA) transporter 1 (GAT-1), and others play important roles in the homeostasis, activation, and differentiation of peripheral naive T cells [64,65]. ASCT2 is a Na^+^-dependent neutral amino acid transporter essential for maintaining cellular glutamine homeostasis and cells’ survival and proliferation dependent on glutamine metabolism [66]. The rapid uptake of glutamine depends on the amino acid transporter ASCT2 and is associated with the activation of naive T cells. LAT1 and ASCT2 are positive regulators of Th1 and Th17 cell differentiation, whereas GAT-1 is a negative regulator of Th1 and Th17 cell differentiation [66]. In a murine experimental autoimmune encephalomyelitis (EAE) model, CD4 T cell-specific deletion of ASCT2 markedly reduced the Th1 and Th17 immune responses and attenuated the progression of EAE.

### 4.2. Glutamine Metabolism in T Cells

Glutamine is the most abundant amino acid in blood serum. It fuels the rapid division of cells such as lymphocytes and is a nitrogen source for T cell activation. Metabolite tracing studies have revealed that activated T cells use glutamine for anaplerosis of α-ketoglutarate (AKG), decreasing the rate of pyruvate entry into the mitochondria in favor of lactate fermentation. AKG acts as a metabolic regulator, determining whether naive T cells differentiate into Th1 or Treg cells [67].

Glutamine can maintain T cell function through TCA cycle metabolism when glucose utilization is limited [68]. In human systemic lupus erythematosus (SLE), the serum glutamine level is significantly higher than that of healthy subjects, negatively associating with the SLE disease activity index (SLEDAI) score. When glutamine levels are restored, the function of the cellular mitochondria is improved in SLE, which triggers a shift from anaerobic metabolism to mitochondrial respiration [69]. Conversely, the tumor environment depends on exogenous glutamine catabolism. Tumor-specific CD8 T cells could effectively eliminate tumors and increase the survival rate of tumor-transplanted mice when cultured under glutamine-limiting conditions or treated with a specific inhibitor of glutamine metabolism [70]. Tumor-infiltrating CD8 T cells showed a reduced surface programmed cell death protein 1 (PD-1) expression and increased Ki67 positivity under glutamine-restricted conditions [70].

### 4.3. Metabolic Features of Other Amino Acids in T Cells

Leucine is an essential amino acid shown to play an important role in the adaptive immune response. Specifically, intracellular leucine tightly regulates the mammalian target of the mTOR signaling pathway and promotes the differentiation of Th1, Th2, and Th17 effector cells [71]. As a rapamycin-like agent, N-acetylleucine amide inhibits cell cycle progression in T cells, resulting in cell cycle arrest in the G1 phase [72].

The uptake of large neutral amino acids (LNAAs) in activated T cells is mediated by a separate L-system (leucine preference system) transporter, SLC7A5, which belongs to the SLC family that transports large neutral and branched-chain amino acids, including leucine [65]. SLC7A5-deficient T cells cannot respond to antigens, clonal expansion, or effector differentiation, resulting in a metabolic alteration that reflects a requirement for continued LNAA-leucine incorporation for activation of the mammalian target of rapamycin complex 1 (mTORC1) and expression of c-Myc [65]. Thus, the availability of intracellular leucine and the activity of mTOR may be critical regulators of T cell differentiation [65].

Tryptophan and arginine can also regulate the activation of T cells. Local T cell tryptophan deficiency can inhibit proliferation, increase T cell apoptosis, and promote differentiation to Tregs. Indoleamine 2,3-dioxygenase (IDO) [73] is an enzyme involved in tryptophan catabolism. When the activity of IDO is inhibited or the gene is silenced, the intracellular tryptophan level will rise, which induces T cells to increase the differentiation of Th1 and Th17 cells. Therefore, blocking the IDO can damage the production of oral antigen-specific Tregs, interfere with the induction of oral tolerance, and aggravate inflammation in the body [74,75,76].

Activation of T cells modulates the enzymes of the serine, glycine, and single-carbon (SGOC) metabolic network, rapidly increasing the processing of serine to single-carbon metabolism. Even in glucose-sufficient conditions, T cell proliferation can be significantly reduced in a medium lacking serine and glycine [77]. Restricting dietary serine impairs pathogen-driven expansion of T cells in vivo without affecting overall immune cell homeostasis. Mechanistically, serine provides glycine and one carbon unit for de novo nucleotide synthesis in T cells [77]. Thus, serine, as a key metabolite of the immune system, directly regulates adaptive immunity by controlling the proliferative capacity of T cells [77].

## 5. Metabolic Adaptations of T Cells in Autoimmune Diseases

### 5.1. Rheumatoid Arthritis

**Figure 3:**
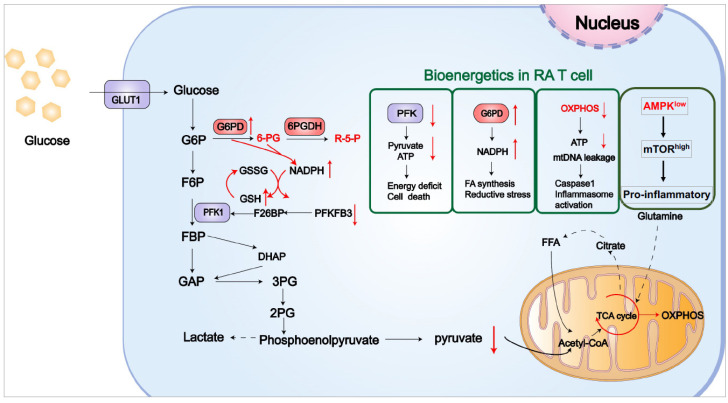
Metabolic adaptations in T cells of RA patients. T cells from RA patients are PFKFB3^low^ and G6PD^high^, shifting the glucose flux from glycolysis into the PPP. Accordingly, these T cells suffer from pyruvate and ATP insufficiency and are sensitive to cell death, while the robust PPP leads to NADPH^high^, facilitating lipogenesis and cell mobility. At the same time, mitochondrial dysfunction in RA T cells drives the release of mtDNA into the cytoplasm, triggering inflammasome-mediated proptosis. In addition, AMPK fails to respond to low ATP in RA T cells, exerting AMPK inactivation and mTOR hyperactivation. The above results can lead topro-inflammatory T cell mal-differentiation in clinical patients.

Rheumatoid arthritis (RA), the most common autoimmune disease in clinical practice, is characterized by chronic, symmetrical, destructive joint inflammation. As shown in Figure 3, there is abnormal energy metabolism in the T cells of RA patients. T cell glycolysis products are reduced in active RA patients, and metabolic adaptations in RA T cells are closely linked to synovial tissue inflammation in these patients. 

Compared to healthy T cells, T cells of RA patients have a defective glycolytic flux due to the deficiency of Fructose-2,6-bisphosphatase 3 (PFKFB3) and the upregulation of glucose-6-phosphate dehydrogenase (G6PD) [78]. The reduction in PFKFB3 leads to decreased glycolysis, while excess G6PD shunts glucose to the PPP, leading to NADPH accumulation and ROS depletion. As a key rate-limiting enzyme in glycolysis, PFKFB3 deficiency in T cells from RA patients assigns them as more sensitive to the restriction of glucose utilization and more prone to undergo cell death [78]. Accordingly, RA patients suffer from a chronic loss of T cells, depletion of T cell stores, and the development of lymphopenia [79]. In contrast, ROS depletion in RA T cells leads to the inactivation of ataxia telangiectasia mutated (ATM), inducing the bypass of the G2/M checkpoint and robust proliferation of T cells in RA patients [80]. In addition, RA T cells harbor insufficient MRE11, leading to telomere damage and pre-mature aging. The mitochondrial loss of MRE11 results in an increased accumulation of cytosolic mtDNA and subsequent proptosis of T cells [81].

While RA T cells are deficient in the glycolytic flux and ATP levels, AMPK, a master energy sensor, is inactivated due to the dysregulated N-myristoylation of AMPKβ and the misallocation of lysosomal AMPK. Consequently, mTOR is hyperactivated in RA T cells, resulting in the mal-differentiation of pro-inflammatory Th1 and Th17 cells and diminished Treg cells in RA patients. Considering the crucial role of IL-17 in synovial inflammation and the destruction of cartilage and bone [73], the imbalance between Th17 and Treg is an essential part of the development of RA [82].

Recent studies in active RA patients have shown that the catabolism rate of lipids is higher than that of healthy people. Changes in total cholesterol and high-density lipoprotein cholesterol in patients with rheumatoid arthritis may be negatively related to inflammation [83]. RA patients’ treatment results in an improved inflammatory state, reduced lipid metabolism, and elevated total low-density lipoprotein (LDL) cholesterol levels. Serum phospholipid fatty acids and sphingomyelin levels are markedly altered between healthy subjects and RA patients. The activity of phospholipase A2 is significantly enhanced in the serum of RA patients [84]. While n-3 polyunsaturated fatty acids (PUFA) are recognized as immunosuppressants, n-3PUFA may also be anti-inflammatory by inducing apoptosis of Th1 cells [85]. In support, symptoms of RA can be effectively relieved using appropriate supplementation with n-3PUFA, and people who consume more than 0.21 g of n-3PUFA per day have a 35% lower risk of RA than those who consume less [86].

### 5.2. Systemic Lupus Erythematosus

**Figure 4:**
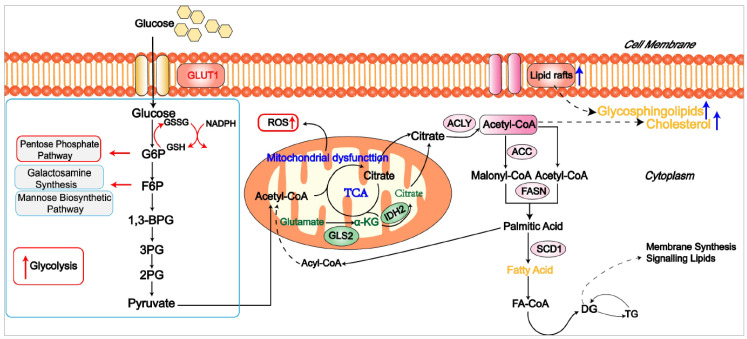
Metabolic features of T cells from SLE patients. Unlike RA T cells, T cells in SLE demonstrate elevated levels of glycolysis and higher levels of mitochondrial ROS. Meanwhile, SLE T cells might also have increased lipid rafts and cholesterol levels, indicating metabolic lipogenesis adaptations.

There are several glucose metabolism abnormalities in the T cells of SLE (Figure 4). Unlike the glycolysis and mitochondrial metabolic defects of RA T cells, the glycolysis and mitochondrial oxidation of SLE patients and mouse T cells are significantly increased, which is related to T cell activation and disease progression. In lupus mice [4], the mitochondrial metabolic inhibitor metformin and glucose metabolism inhibitor 2-deoxy-G-glucose (2-DG) have high levels of interferon-γ, which effectively reduce the production of ECAR, OCR, and IFN-γ of T cells [4,87]. These results suggest that a promising therapeutic approach for SLE may be normalizing T cell metabolism with dual inhibition of glycolysis and mitochondrial metabolism. Meanwhile, PPP metabolites, such as R5P and F6P, are higher in peripheral blood lymphocytes from SLE patients, suggesting that activation of T cells in SLE patients involves three significant pathways of glucose metabolism: aerobic glycolysis, PPP, and mitochondrial oxidative phosphorylation [88].

GLUT1 and recombinant human calmodulin-dependent protein kinase (CaMK4) expression levels are significantly elevated in effector T cells from patients with active SLE compared with healthy controls [89]. Functional studies demonstrate that the inhibition of CaMK4 can reduce the expressions of GLUT1 and IL-17 during the differentiation of Th17 cells. Further, overexpression of GLUT1 in T cells leads to lupus-like disease and the selective accumulation of effector and follicular T cells [89,90]. In spontaneous and induced SLE mouse models, the GLUT1 inhibitor CG-5 can reduce glucose intake, improve the autoimmune phenotype, and effectively alleviate disease symptoms. By inhibiting Th1 and Th17 differentiation and promoting Treg cell induction, CG-5 regulates CD4 T cell polarization. Thus, inhibiting glycolysis of T cells is promising for improving autoimmune SLE [91].

Lipid metabolism is essential in biological processes and cellular functions. It is involved in developing SLE (Figure 4). SLE patients have abnormal lipid metabolism of peripheral blood mononuclear cells (PBMCs) compared with normal controls. Specifically, SLE patients’ cholesterol and sphingolipid homeostasis in T cells are changed. Sphingolipid synthesis is significantly increased, leading to the increase in lipid raft formation and the dysfunction of T cells [92]. These changes in lipids can be corrected after in vitro antioxidant culturing of PMBCs from SLE patients. Correction of abnormal lipid metabolism can inhibit the autoimmune response of SLE PBMCs and reduce increased oxidative stress [92]. N-butyldeoxynojirimycin (NB-DNJ), a glycosylceramide synthase inhibitor, has recently been shown to restore intracellular trafficking and normal lipid metabolism within T cells in SLE patients. NB-DNJ treatment can restore the lipid metabolism of lupus T cells and further inhibit their proliferation [93]. Glycosphingolipids, consisting of a nonlipid glycosyl moiety linked to a sphingosine moiety, affect cell signal transduction. Glycosphingolipids are abundantly present in the kidney and have been implicated in the pathogenesis of lupus nephritis. In patients with lupus nephritis and MRL/LPR lupus mice, glycosphingolipid metabolism is functionally abnormal [94]. The levels of glycosylceramide (Glcer) and lactosylceramide (LacCer) in the kidney are markedly increased, which may serve as early markers of lupus nephritis [94]. Inhibiting glycosphingolipid synthesis significantly inhibits in vitro T cell activity and anti-dsDNA antibody production in SLE patients [95]. As lipid rafts are platforms for the well-ordered accumulation of cholesterol and sphingomyelin, changes in the composition and dynamics of lipid rafts in SLE T cells may affect the TCR signaling and thus may be promising therapeutic targets in clinical practice [96].

### 5.3. Multiple Sclerosis

The conversion of phosphoenolpyruvate to pyruvate during glycolysis is catalyzed by pyruvate kinase (PK), whose isoform is PKM2. PKM2 regulates Th17 cell differentiation and autoimmune inflammation of EAE [97]. Specifically, PKM2 is highly exposed during the differentiation and development of Th17 cells in EAE. PKM2-deficient T cells impair their ability to differentiate into Th17 cells, thereby reducing Th17-mediated inflammation and ameliorating the clinical symptoms of EAE. Mechanistically, PKM2 can translocate into the nucleus and interact with STAT3, enhancing STAT3 activity and thus increasing Th17 cell differentiation. In addition, PKM2 is upregulated upon in vitro activation of mouse and human CD4 T cells, and PKM2 is capable of phosphorylation and accumulates in the nucleus [98], serving as a potential target for treating T cell-mediated autoimmunity. In addition, glucosamine, an amino sugar, exhibits immunosuppressive effects on IL-2-mediated T cell proliferation and cytokine release [99,100], negatively affecting EAE [101,102]. Glucosamine also strongly inhibits the differentiation of Th1 cells and ameliorates the severity of EAE [103].

Removing mitochondrial ROS (mtROS) from Treg in EAE mice can prevent Treg from dying, while weakening Th1 and Th17 autoimmune responses, emphasizing the thankless role of mitochondrial oxidative stress in defining the fate of Treg in the autoimmune process [104]. And, while the glycolytic gene Gpi1 is selectively required by inflammation-causing Th17 cells but not by homeostatic Th17 cells, deleting Gpi1 selectively eliminates inflammatory brain-derived and colonic inflammatory Th17 cells without substantially affecting homeostatic microbiota-specific Th17 cells [105]. Thus, inhibition of glycolysis by targeting Gpi1 may be an effective therapeutic strategy for Th17-mediated autoimmune diseases [105]. Further, the inactivation of mitochondrial DRP1 with a small-molecule inhibitor, Mdivi-1, can reduce the severity of EAE by reducing the number of Th1 and Th17 cells and elevating the number of Foxp3^+^ Treg cells in the central nervous system (CNS) [106].

FABPs are intracellular fatty acid carrier proteins that play an essential role in the intracellular utilization of fatty acids. Mice deficient in FABPs, especially epidermal-type FABP (E-FABP), are protected against the development of EAE [107]. Further, E-FABP-deficient T cells exert enhanced expression of peroxisome proliferator-activated receptor γ (PPARγ), which can prevent Th17 differentiation and improve the generation of Tregs [107]. Accordingly, EI-03, a novel E-FABP inhibitor that binds to the lipid-binding pocket of E-FABP [60], increases the expression of PPARγ, blocks the effector T cell differentiation, and thus ameliorates EAE. In addition, apolipoprotein E (apoE), an essential player in lipid transport and cholesterol metabolism, may be correlated with the disease severity of MS. ApoE deficiency increases the proportion of Th1 and Th17 cells in the spleen and brain. At the same time, apoE mimetic peptide CDG112-treated mice exhibit lower Th1 and Th17 cell frequencies and reversed adverse effects of EAE [108].

ATF4, a primary leucine zipper transcription factor, is a member of the ATF/CREB protein family [109]. ATF4 mRNA is widely expressed and can be induced in T cells under various conditions [110]. ATF4 provides precursors and energy for anabolic pathways by promoting glycolysis, glutamine catabolism, and oxidative phosphorylation. In ATF4-deficient mice [104], the immune response of Th1 cells is reduced, and the immune response of Th17 cells is elevated, contributing to more severe symptoms of EAE.

### 5.4. Other Autoimmune Diseases

#### 5.4.1. Psoriasis

Psoriasis is a common chronic, intermittent autoimmune disease of the skin and joints. Chronic, symmetrically erythematous, or scaly papules are the most common feature of psoriasis patients. T cells are essential in disease pathogenesis [111,112], driving permanent auto-inflammation [113].

Glutaminolysis, an abnormal activation mediated by glutaminase 1 (GLS1), promotes Th17 and γδ T17 (IL-17A-producing γδ T cell) cell differentiation by enhancing histone H3 acetylation of the IL17A promoter, leading to an immune imbalance and the development of psoriasis [114]. Mechanistically, mucosa-associated lymphoid tissue lymphoma translocation protein 1 (MALT1) protease is constitutively active in CD4 T cells and γδ T cells in psoriasis, and MALT1 can stabilize the binding of c-Jun to the GLS1 promoter region, thereby promoting the expression of GLS1 [114]. Accordingly, GLS1 or the MALT1 protease activity blockade attenuates Th17 and γδ T17 cell differentiation and epidermal proliferation in a psoriasis-like mouse model. In the collective, the MALT1/cJun/GLS1/glutaminolysis/H3 acetylation/T17 axis is involved in psoriasis pathogenesis and may represent a potential therapeutic target for psoriasis [114].

#### 5.4.2. Type 1 Diabetes

Type 1 diabetes (T1D) is a metabolic syndrome characterized by a loss of tolerance to pancreatic β-cells and defective Treg function, leading to hyperglycemia [115]. In T1D, pancreatic β-cells suffer from autoimmune attacks by T cells [116]. Specifically, T cells undergo metabolic reprogramming from oxidative phosphorylation to aerobic glycolysis during T cell activation. In contrast, reduced mTOR activation and reduced conversion to aerobic glycolysis are observed in diabetic splenocytes using scavenging ROS and the potent antioxidant manganese metalloporphyrin (MnP) [117]. Therefore, interfering with ROS-mediated redox regulation hinders the reprogramming of T cell metabolism to glycolysis and reduces the activation of autoreactive T cells, which may delay the progression of diabetes [116]. PFK15, a competitive inhibitor of the rate-limiting glycolytic enzyme PFKFB3 [118], inhibits glycolytic utilization of diabetic CD4 T cells in vitro and reduces T cell responses to β-cell antigens, delaying the onset of diabetes in a T1D model [115]. Glycolytic inhibition eventually depletes diabetic CD4 T cells, which is irreversible in vitro and in vivo. Together, inhibition of glycolysis drives T cell depletion, and metabolic regulation may serve as a new therapeutic target for controlling T cell metabolism and restoring autoimmune tolerance [119].

Mitochondrial inner membranes in T cells of T1D patients exert hyperpolarization (MHP), whereas MHP is not elevated in T cells of type 2 diabetic patients. Functional studies provide evidence that activation-induced IFN-γ production in T cells from T1D patients after TCR stimulation is responsible for the changes in T cell MHP [120], resulting in lower levels of intracellular ATP. Thus, intrinsic mitochondrial dysfunction of T cells in T1D alters their mitochondrial capacity to produce ATP and IFN-γ, affecting T cell bioenergetics and function [120].

D-mannose, a C-2 heterodimer in human blood at concentrations below 1/50 glucose, induces the differentiation of human and mouse naive CD4 T cells to Treg cells by promoting TGF-β activation with the involvement of ROS [121,122]. Consistently, the pathological changes and airway inflammation in autoimmune diabetic mice are suppressed by d-mannose supplementation, serving as a promising application in clinical medicine [122].

#### 5.4.3. Sjögren’s Syndrome

Sjögren’s syndrome (SS) is a chronic inflammatory autoimmune disease primarily affecting exocrine glands. The clinical manifestations are salivary and lacrimal gland damage, accompanied by the production of a variety of autoantibodies in the circulation [123,124]. Mechanistically, SS is characterized by over-proliferation and hyperactivation of salivary CD4 T cells. Knockdown of the lncRNA PVT1 gene can reduce the proliferation of T cells in SS patients, accompanied by a lower expression of glycolytic genes and essential glycolytic proteins directly transcribed by Myc [125]. Functional studies reveal that PVT1-deficient T cells have defective lactate secretion and glucose uptake. Thus, upregulating the lncRNA PVT1 in CD4 T cells of SS patients maintains Myc expression and controls CD4 T cell proliferation and effector functions by regulating the reprogramming of glycolysis [125]. While glycolysis is increased in CD4 T cells from SS-like NOD/Ltj mice, treating diseased mice with the glycolysis inhibitor 2-DG significantly reduces the extent of lymphocyte infiltration and CD4 T cells in submandibular gland lesions, improving impaired salivary gland flow [126]. In addition, GLS1, a critical factor in glutamine catabolism, is upregulated in infiltrated labile CD4 T cells and circulating CD4 T cells from SS patients. The selective glutaminase variant inhibitor bis-2-(5-phenylacetamido-1,2,4-thiadiazol-2-yl)ethyl sulfide (BPTES) downregulates ECAR and OCR levels of activated CD4 T cells in SS mice [126], reducing lymphocyte infiltrates and thus restoring salivary flow. Therefore, GLS1 might have a positive association with the development of SS. BPTES inhibition of GLS1 normalizes the metabolic state and effector functions of CD4 T cells and effectively alleviates SS symptoms [126].

Metformin, an inhibitor for mitochondrial respiratory chain complex I, activates AMPK and inhibits mTOR, exerting anti-inflammatory activity [127]. Thus, metformin enhances Treg cells and downregulates Th1 and Th17 cells, effectively improving salivary gland function in SS models [128,129]. In addition, sphingosine 1-phosphate (S1P) significantly promotes the production of IFN-γ by CD4 T cells in primary SS patients compared to healthy controls, and modulating the S1P1 signaling pathway may be a novel strategy for the treatment of primary SS [130].

## 6. Conclusions

As an important immune cell population, T cells participate in immune regulation and play an important role in maintaining homeostasis. While T cells use metabolites to provide energy, different T cell subsets have different metabolic pathways, which can, in turn, regulate their differentiation and function. Under autoimmune conditions, the metabolic status of T cells changes accordingly. Targeting metabolic reprogramming of T cells can shift the differentiation and functional status of T cells in autoimmune diseases. Meanwhile, mitochondria, as the core sites of metabolism, play a central role in the metabolic reprogramming of T cells. Exploring the functions of mitochondria and metabolism reprogramming in T cell-mediated autoimmune diseases may provide novel insights for the in-depth understanding of disease pathogenesis and the development of targeted drugs, optimizing the clinical management of patients.

## Data Availability

Not applicable.
